# Climate constrains the evolutionary history and biodiversity of crocodylians

**DOI:** 10.1038/ncomms9438

**Published:** 2015-09-24

**Authors:** Philip D. Mannion, Roger B. J. Benson, Matthew T. Carrano, Jonathan P. Tennant, Jack Judd, Richard J. Butler

**Affiliations:** 1 Department of Earth Science and Engineering, Imperial College London, London SW7 2AZ, UK; 2 Department of Earth Sciences, University of Oxford, Oxford OX1 3AN, UK; 3 Department of Paleobiology, National Museum of Natural History, Smithsonian Institution, PO Box 37012, Washington DC 20013-7012, USA; 4 School of Geography, Earth and Environmental Sciences, University of Birmingham, Birmingham B15 2TT, UK

## Abstract

The fossil record of crocodylians and their relatives (pseudosuchians) reveals a rich evolutionary history, prompting questions about causes of long-term decline to their present-day low biodiversity. We analyse climatic drivers of subsampled pseudosuchian biodiversity over their 250 million year history, using a comprehensive new data set. Biodiversity and environmental changes correlate strongly, with long-term decline of terrestrial taxa driven by decreasing temperatures in northern temperate regions, and biodiversity decreases at lower latitudes matching patterns of increasing aridification. However, there is no relationship between temperature and biodiversity for marine pseudosuchians, with sea-level change and post-extinction opportunism demonstrated to be more important drivers. A ‘modern-type' latitudinal biodiversity gradient might have existed throughout pseudosuchian history, and range expansion towards the poles occurred during warm intervals. Although their fossil record suggests that current global warming might promote long-term increases in crocodylian biodiversity and geographic range, the 'balancing forces' of anthropogenic environmental degradation complicate future predictions.

## Abstract (web summary)

Crocodylians and their relatives have a rich evolutionary history. Here the authors show long-term decline of terrestrial crocodylians driven by decreasing temperatures but no relationship between temperature and biodiversity for marine crocodylians over their 250 million year history.

## Graphical abstract

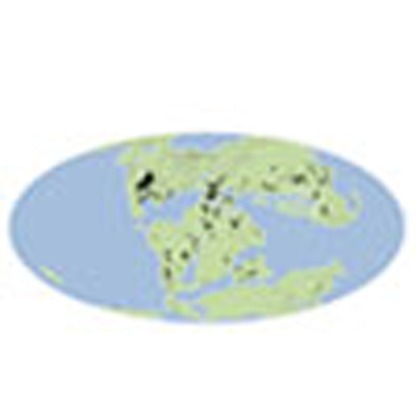

Ongoing climate change, with projected global warming of 2.0–4.8°C over the next century^1^, could have profound repercussions for crocodylian distributions and biodiversity. As ectotherms, living crocodylians are environmentally sensitive^2^, and 10 of the 23 extant species are at high extinction risk (http://www.iucncsg.org/; 2015). Ecological models can predict the responses of extant species distributions to rising temperatures. However, only the fossil record provides empirical evidence of the long-term interactions between climate and biodiversity^3^, including during intervals of rapid climate change that are potentially analogous with the present^4^.

Pseudosuchia is a major reptile clade that includes all archosaurs closer to crocodylians than birds, and made its first fossil appearance nearly 250 million years ago, at the time of the crocodile–bird split^5^. Crocodylians, the only extant pseudosuchians, are semi-aquatic predators with low morphological diversity, and a tropically restricted geographic range (a band of ∼35° either side of the Equator)^6^. Although often regarded as ‘living fossils', the pseudosuchian fossil record reveals a much richer evolutionary history and high ancient biodiversity. This poses a key question about the drivers of long-term evolutionary decline in groups that were highly diverse in the geological past, especially given the extraordinary high biodiversity (∼10,000 species) of the only other extant group of archosaurs, birds^7^. Over 500 extinct pseudosuchian species are known (this study), with a broader latitudinal distribution^6^ and a wider array of terrestrial ecologies than their living counterparts^8^,^9^. Several diverse lineages also independently invaded marine environments^10^. Body plans and feeding modes showed much higher diversity, including flippered taxa and herbivorous forms^10^,^11^,^12^,^13^, and body sizes varied from dwarfed species <1 m in length^14^, to giants such as *Sarcosuchus*, which reached around 12 m in length and weighed up to 8 metric tons^15^. Climate has often been proposed to have shaped pseudosuchian biodiversity through time^6^,^8^,^16^, and the group's geographic distribution over the past 100 million years has been used as evidence in palaeoclimatic reconstructions^2^.

Here we examine the effect of climate on spatiotemporal patterns in pseudosuchian biodiversity over their 250 million year history, using a comprehensive fossil occurrence data set. Our study is the first to analyse climatic drivers of pseudosuchian biodiversity through the group's entire evolutionary history, applying rigorous quantitative approaches to ameliorate for uneven sampling across both time and space. Furthermore, this is the only comprehensive, temporally continuous fossil occurrence dataset for a major extant vertebrate group: equivalent datasets are not currently available for mammals, birds, squamates, teleosts, or other groups with evolutionary histories of similar durations.

## Results and discussion

### The fossil record at face value

**Figure 1:**
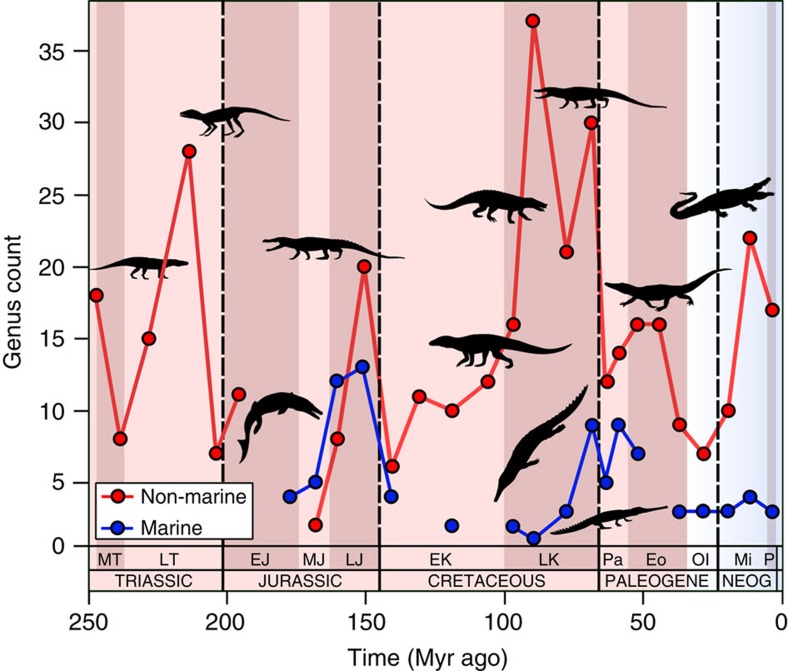
Global raw counts of pseudosuchian genera through the last 250 million years. Red line represents non-marine taxa and the blue line represents marine taxa. Silhouettes of representative pseudosuchians are modified from work by Dmitry Bogdan, Evan Boucher, Scott Hartman, Mike Keesey, Nobumichi Tamura (hosted at: http://phylopic.org, where all license information is available).

**Figure 2:**
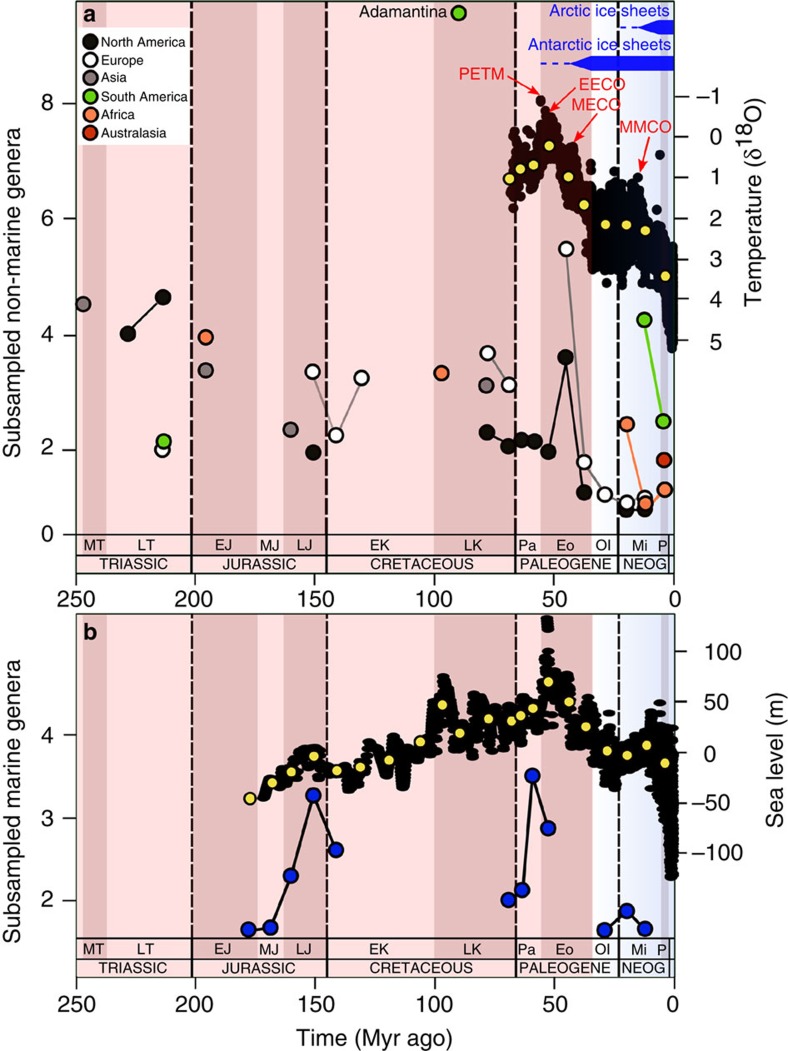
Subsampled pseudosuchian genus biodiversity. (**a**) Non-marine biodiversity within continental regions for a subsampling quorum level of 0.4, and palaeotemperature (*δ*^18^O) curve for the last ∼70 Myr ago^17^ (with weighted means (yellow circles)). (**b**) Global marine biodiversity for a subsampling quorum level of 0.4 (blue circles) and sea level curve^18^ (with weighted means (yellow circles)). Adamantina, Adamantina Formation subsampled crocodylomorph biodiversity; EECO, Early Eocene Climatic Optimum; MECO, Mid-Eocene Climatic Optimum; MMCO, Mid-Miocene Climatic Optimum; PETM, Paleocene-Eocene Thermal Maximum. Linear regression for pooled regional subsampling results on geological age in millions of years: log_10_ subsampled genera=0.0015 × age+0.244 (*P*=0.002; *N*=38; *R*^2^=0.221).

An uncorrected global census of pseudosuchian genera (Fig. 1; and species, Supplementary Fig. 1) shows an apparent trend of increasing biodiversity through the Mesozoic, punctuated by a latest Triassic crash, a severe decline across the Jurassic/Cretaceous (J/K) boundary and the Cretaceous/Paleogene (K/Pg) mass extinction, from which recovery only started in the early Neogene. However, application of sampling standardisation reveals a different and more nuanced story (Fig. 2).

### Biodiversity in the early Mesozoic

**Figure 3:**
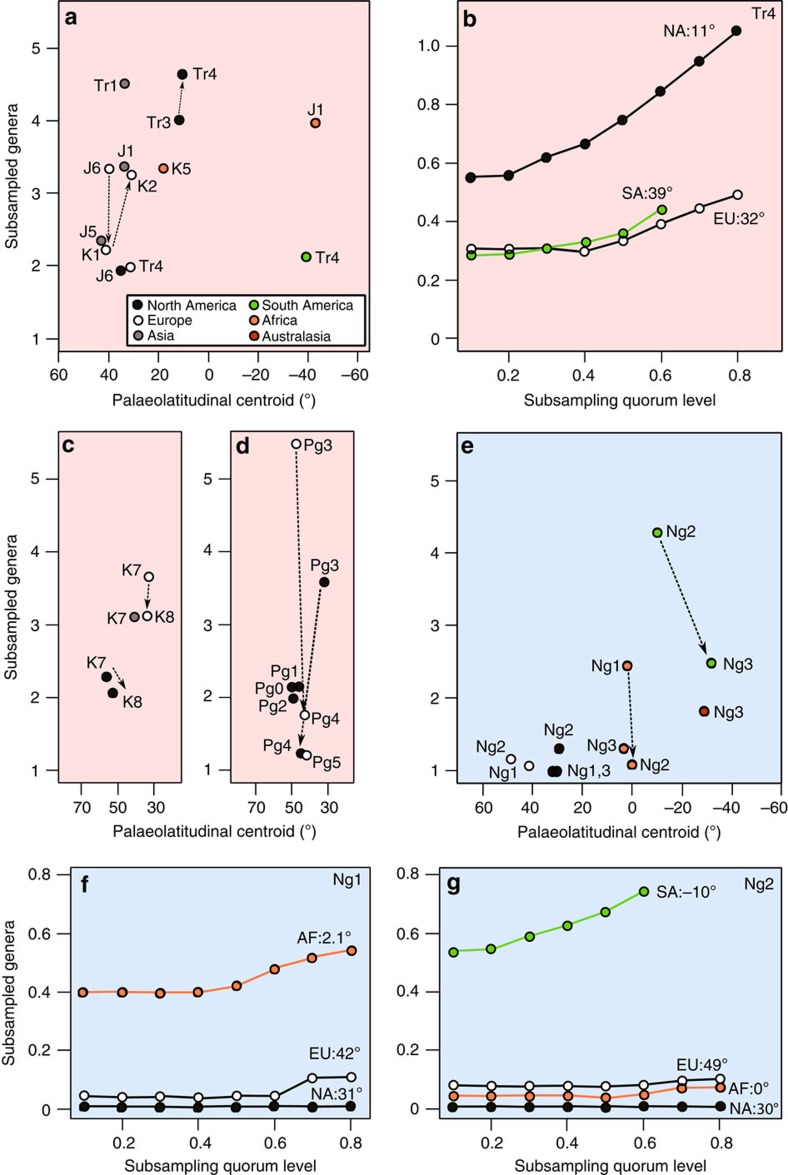
Regional plots of subsampled biodiversity versus palaeolatitudinal centroid and subsampling curves for non-marine genera. (**a**) Triassic–early Late Cretaceous (K6) subsampled biodiversity versus palaeolatitudinal centroid. (**b**) Late Triassic (Tr4) log_10_-transformed subsampling curves. (**c**) Late Cretaceous (K7 and K8) subsampled biodiversity versus palaeolatitudinal centroid. (**d**) Paleogene subsampled biodiversity versus palaeolatitudinal centroid. (**e**) Neogene subsampled biodiversity versus palaeolatitudinal centroid. (**f**) Early Neogene (Ng1) log_10_-transformed subsampling curves. (**g**) Middle Neogene (Ng2) log_10_-transformed subsampling curves. Dashed arrows denote notable changes in biodiversity between consecutive time intervals within a single continental region. K, Cretaceous; Ng, Neogene; Tr, Triassic.

Subsampled pseudosuchian biodiversity reached a peak during the earliest intervals of the group's history and shows a long-term pattern of gradual decline towards the present day (Fig. 2). Substantial short-term volatility around this trend, indicated by a low coefficient of determination (linear regression: *N*=38, *R*^2^=0.221; Fig. 2), suggests that this overall pattern was punctuated by the extinctions and radiations of individual clades (see below). Nevertheless, for much of the Triassic, non-marine pseudosuchian biodiversity exceeded that of nearly all later time intervals (Fig. 2a), indicating exceptionally rapid early diversification^19^. We also find evidence for a strong palaeotropical biodiversity peak during the Late Triassic (Fig. 3a,b), similar to the modern-day latitudinal biodiversity gradient^20^.

Only the crocodylomorph clade survived the Triassic/Jurassic mass extinction (201 Myr ago)^5^. During the Jurassic, non-marine crocodylomorph biodiversity remained depressed relative to the Triassic pseudosuchian peak (Fig. 2a). However, the group radiated into new morphospace^21^, and thalattosuchian crocodylomorphs invaded the marine realm by the late Early Jurassic^10^,^12^. Subsequently, marine crocodylomorph biodiversity increased until at least the Late Jurassic^16^ (Fig. 2b), tracking a general trend of rising eustatic sea levels^22^. The earliest Cretaceous fossil record is considerably less informative than those of many other intervals^23^. However, our subsampled estimates are congruent with observations of phylogenetic lineage survival^16^,^24^,^25^, and indicate that both marine and non-marine crocodylomorph biodiversity declined across the J/K boundary (Figs 2 and 3a), including the extinction of teleosauroid thalattosuchians^16^,^25^.

**Figure 4:**
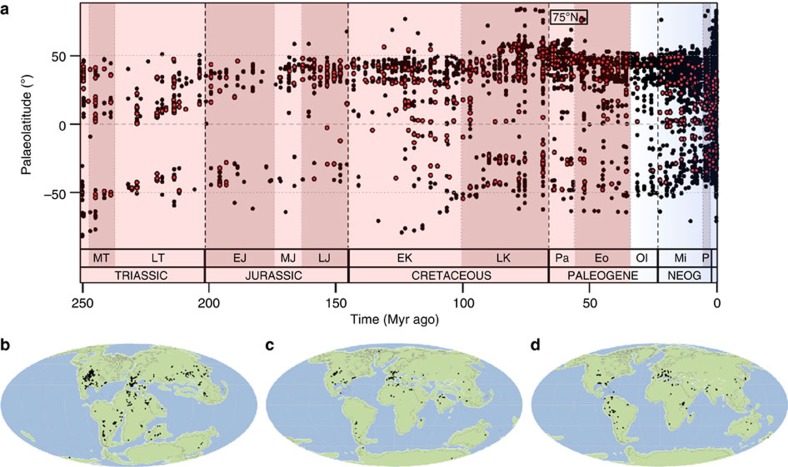
Palaeolatitudinal distribution of Pseudosuchia. (**a**) Palaeolatitudinal spread of all non-marine pseudosuchian (red circles) and all non-marine tetrapod (black circles) occurrences through time. (**b**–**d**) Global palaeocontinental reconstructions from Fossilworks showing the distribution of all pseudosuchian occurrences in the (**b**) Cretaceous (145–66 Myr ago), (**c**) Eocene (56–33.9 Myr ago) and (**d**) Miocene (23–5.3 Myr ago).

Sampling of palaeotropical non-marine crocodylomorphs is limited throughout the Jurassic–Cretaceous (Figs 3a and 4a). However, good sampling of terrestrial early Late Cretaceous North African crocodylomorphs inhabiting a low-latitude (18°N), semi-arid biome^8^,^14^ (Fig. 4b) indicates subsampled biodiversity levels comparable to those of palaeotemperate regions in other Cretaceous time slices (Fig. 2a). Furthermore, sub-palaeotropical (24–28°S) South American crocodylomorphs of the Late Cretaceous Adamantina Formation were exceptionally diverse^8^,^26^, raising the possibility that pseudosuchians in fact reached their highest biodiversities in tropical environments during the mid-Cretaceous greenhouse world. In contrast to previous work^6^, this suggests that there was no palaeotropical trough in Cretaceous crocodylomorph biodiversity, differing from the pattern recovered for contemporaneous dinosaurs^27^. Previous work on both crocodylomorphs^6^ and dinosaurs^27^ found low palaeotropical biodiversity by effectively ‘averaging' global biodiversity across palaeolatitudinal bands, applying sampling standardization indiscriminately, regardless of the distribution of data quality. In contrast, here we used an approach in which regional biodiversities were estimated only when sufficient data were available to do this reliably, using a coverage estimator (see Methods). New and improved data, coupled with more appropriate methods, likely explains the differences between our results and that of previous work on crocodylomorphs^6^, but it remains to be seen whether the difference with the dinosaurian pattern^27^ is genuine.

### Biodiversity across the K/Pg boundary

The Cretaceous witnessed the non-marine radiations of notosuchians^8^,^19^,^26^ and eusuchians, with crocodylians diversifying from within Eusuchia during the Late Cretaceous (Santonian–Maastrichtian)^9^,^28^. Despite this, subsampled non-marine biodiversity decreased from the Campanian into the Maastrichtian in both Europe and North America (Figs 2a and 3c), on the ∼9 million-year timescale resolution of our study. This decrease in biodiversity prior to the K/Pg mass extinction event (66 Myr ago) mirrors the pattern seen in North American mammals^29^ and some dinosaur groups^30^. However, rather than signalling that a protracted global catastrophe caused the K/Pg mass extinction, this latest Cretaceous decline of crocodylomorphs tracks a long-term trend towards cooler temperatures through the Late Cretaceous^31^,^32^. This is consistent with the ‘background' coupling between biodiversity and global climate observed throughout the late Mesozoic and Cenozoic (see below), and likely characteristic of the entire evolutionary history of Pseudosuchia.

The effect of the K/Pg mass extinction on crocodylomorphs has previously been perceived as minor or non-existent^19^,^28^, with any extinction temporally staggered^33^. However, several non-marine groups with high biodiversity before the boundary became extinct (most notably all non-sebecid notosuchians^34^), and only two clades (the marine dyrosaurids and terrestrial sebecids) survived alongside crocodylians^28^,^35^. Nevertheless, the extinctions of these groups, and other non-marine crocodylomorph taxa were balanced by rapid radiations of the three surviving clades in the early Paleocene^19^,^28^,^34^,^36^, including substantial range expansions of marine dyrosaurids^36^,^37^ and terrestrial alligatoroids^28^ into South America. Range expansions and increases in regional taxon counts among dyrosaurids^35^,^36^,^37^ and gavialoid crocodylians^38^ led to a substantial increase in global marine crocodylomorph biodiversity by the late Paleocene (Fig. 2c), with crocodylomorphs potentially benefiting from the extinction of many other marine reptiles at the K/Pg boundary^36^,^39^.

### Non-marine biodiversity and palaeotemperature

Relative changes in subsampled non-marine biodiversity in both North America and Europe track each other and the *δ*^18^O palaeotemperature proxy^17^,^40^ through the Cenozoic (Fig. 2a; Table 1). The relationship between these variables is characterised by near-zero, negative serial correlation for North American data, and high, negative serial correlation for European data. The occurrence of near-zero estimated serial correlation suggests links between high amplitude, long-term patterns, with weaker correspondence between low amplitude, short-term fluctuations. Nevertheless, a similar relationship is still recovered when serial correlation is assumed to equal zero, and for the European data when it is assumed to equal one (Table 1), demonstrating robustness of this result to statistical approach. Furthermore, the recovery of near-identical patterns of relative standing biodiversity from separate European and North American occurrence datasets suggests that our subsampling approach is effective in recovering a shared underlying biodiversity pattern.

### Paleogene non-marine biodiversity

These North American and European patterns indicate that non-marine crocodylomorphs remained diverse at temperate palaeolatitudes (30–60°) during the early Paleogene greenhouse world (66–41 Myr ago). There is no evidence for transient biodiversity increases driven by the short-term Paleocene–Eocene Thermal Maximum (56 Myr ago), possibly because the timescale of species origination and phenotypic divergence that would allow speciation to be recognisable in the fossil record is longer than that of this rapid climatic event (>5 °C in <10,000 years^4^). Nevertheless, early Eocene crocodylomorphs expanded their palaeogeographic range to at least 75°N^7^ (Fig. 4a,c), coinciding with the sustained high temperatures of the Early Eocene Climatic Optimum (53–50 Myr ago)^41^. A major European and North American biodiversity peak during the middle Eocene (48–41 Myr ago; Fig. 2a) is composed primarily of crocodylians, with sebecids, previously known only from South America^34^, also present in Europe^42^. However, although this interval includes the short-term hyperthermal Mid-Eocene Climatic Optimum, the overall trend is one of cooling^17^, indicating a temporary decoupling of temperature and biodiversity. At temperate palaeolatitudes, a stark late Eocene–Oligocene (41–23 Myr ago) decline to unprecedentedly low biodiversity (Figs 2a and 3d) coincides with global cooling, the development of a strengthened latitudinal temperature gradient^43^, and the onset of Antarctic glaciation^17^. Unfortunately, southern hemisphere and palaeotropical (0–30°) sampling (Fig. 3d) is inadequate to determine additional patterns of Paleogene biodiversity, including the form of palaeolatitudinal biodiversity gradients. This also means that we cannot determine whether the correlation between palaeotemperature and non-marine biodiversity was restricted to northern temperate palaeolatitudes, or was a global pattern, during the Paleogene. If the latter is shown to have been the case, then we should ultimately expect to find extremely high Paleogene biodiversity in currently poorly sampled regions such as South America. Alternatively, temperature change might drive pseudosuchian biodiversity only at limiting, low–medium temperatures. At high, non-limiting temperatures, other factors such as aridity might become limiting, as suggested by low-latitude Cenozoic biodiversity patterns described below.

### Cenozoic marine biodiversity

Marine crocodylomorph biodiversity decreased in the early Eocene (Fig. 2b), with the loss of basal gavialoids (‘thoracosaurs') and decline in dyrosaurids, with the latter group becoming extinct in the middle–late Eocene^16^,^28^,^37^. This observation conflicts with the conclusions of a recent study that did not use subsampling approaches^16^, the authors of which proposed that marine crocodylomorphs generally diversified during warm intervals. Furthermore, contrary to the findings of those authors^16^, there is no statistical relationship between the *δ*^18^O palaeotemperature proxy and marine crocodylomorph biodiversity in any of our analyses, whether or not subsampling is applied (Table 2). This differs from the approach of Martin *et al.*^16^, who found correlations between their palaeotemperature proxy and marine crocodylomorph biodiversity, but only once metriorhynchoid thalattosuchians were excluded. They used this finding as evidence for an assertion that metriorhynchoids had a distinct biology from other marine crocodylomorphs. However, a more conservative reading of these results is that marine crocodylomorph biodiversity was not consistently linked to temperature over the studied interval.

Instead, our analyses find strong, significant relationships between subsampled marine genus counts and eustatic sea level estimates of Miller *et al.*^18^ when including a ‘phase' variable (see Methods) to distinguish the amplitude of thalattosuchian biodiversity patterns from that of stratigraphically younger marine radiations (Table 2). This regression model explains more than 60% of the variance in subsampled marine biodiversity. Directly counted marine genera have a marginally significant relationship with sea level (Table 2). The negative slopes of the ‘phase' variable in these regression models indicate that thalattosuchians attained higher biodiversities relative to sea level than did stratigraphically younger marine crocodylomorphs. Our results support previous observations that continental flooding, through eustatic sea level change, shaped the evolution of marine shelf biodiversity^44^,^45^, including that of near-shore marine reptiles^22^. Other extrinsic factors might also be important; for example, it is likely that post-extinction opportunism contributed to high biodiversity of early Paleogene marine crocodylomorphs, which show a substantial post-extinction biodiversity increase that was facilitated by inter-continental range expansion^36^,^39^. Marine crocodylomorph biodiversity remained low through the remainder of the Paleogene and early Neogene (Fig. 2b), comprising a small number of gavialoids^46^ and tomistomines^28^,^47^, before their present day restriction to non-marine environments.

### Neogene non-marine biodiversity

Temperate palaeolatitudinal biodiversity remained low among Neogene non-marine crocodylomorphs, although a minor peak might be coincident with the Mid-Miocene Climatic Optimum (15 Myr ago; Fig. 2a). There is clear evidence for latitudinal range contraction through time both on the continents (Fig. 4) and in the marine realm (Supplementary Fig. 5). The most poleward crocodylomorph occurrences declined to their approximate present day limits (35° N and S) by the late Miocene (Fig. 4a,d), coincident with the onset of Arctic glaciation^17^. This is despite the occurrence of non-crocodylomorph-bearing fossil localities documenting higher palaeolatitude tetrapod faunas, and indicates that crocodylomorph range contraction is not a sampling artefact.

Neogene terrestrial biodiversity of crocodylians was substantially higher in the palaeotropics than in temperate regions (Fig. 2a), with sufficient data to demonstrate a palaeotropical peak from the early Miocene (Fig. 3e–g). High palaeotropical biodiversity in the middle–late Miocene is linked to the timing of the rapid radiation and dispersal of *Crocodylus*^48^ and other crocodyloid lineages^49^, and the presence of highly diverse sympatric assemblages of crocodylians in the proto-Amazonian mega-wetlands of South America^50^,^51^. Nevertheless, palaeotropical crocodylomorph biodiversity declined in the late Miocene of Africa (Figs 2a and 3e), coincident with the formation of the Sahara Desert^52^ and sub-Saharan expansion of savannah environments^53^. A similar decline ensued in the post-Miocene palaeotropics of South America (Figs 2a and 3e), and has been attributed to hydrographic changes and the disappearance of the mega-wetlands^52^,^53^, driven by Andean uplift^54^. The overall dwindling of crocodylomorph biodiversity towards the present day tracks the late Cenozoic cooling trend^17^, increasing aridification^52^ and the rising predominance of grassland ecosystems during the late Neogene^53^, the Quaternary Ice Ages^17^ and presumably the more recent impact of human activity.

### The future of crocodylians

Our findings show that the biodiversity of non-marine pseudosuchians has been strongly linked to both spatial and temporal temperature variation, as well as the spatial distribution of aridity, throughout the group's evolutionary history. This can be demonstrated most clearly during the Cenozoic, where the long-term decline of crocodylomorphs at temperate latitudes over the last 50 million years has been driven by the descent into the modern-day icehouse world, and the geographic pattern of decline among palaeotropical taxa in the Neogene matches patterns of aridification in Africa and South America. As the Earth continues to warm, perhaps heading towards a greenhouse world comparable to that of the early Paleogene^4^, we might therefore expect that higher temperatures should promote long-term increases in crocodylian biodiversity and the expansion of the group's latitudinal range outside of the tropics, as was the case for much of their Mesozoic and early Cenozoic history. However, in contrast to these earlier times, predictions of the distribution of their future biodiversity are complicated by the impact of human activity on habitat loss and fragmentation, which are likely to reduce the rate and magnitude of crocodylian range expansion^1^, especially into populated regions.

## Methods

### Pseudosuchian occurrences data set

Following extensive work to ensure that occurrences and taxonomic opinions were consistent and up-to-date with the literature^55^, the Paleobiology Database (PaleoDB; http://paleobiodb.org) includes a near-comprehensive dataset of all published pseudosuchian occurrences spanning the Middle Triassic through to the Pleistocene, a period of nearly 250 million years. Pseudosuchian body fossil occurrences that could be assigned to genera (including qualifiers such as cf. and aff.) were downloaded from this database (comprising 2,767 fossil occurrences representing 386 genera), accessed via Fossilworks (http://fossilworks.org), on 23 February 2015.

Genera were used so as to incorporate specifically indeterminate material, enabling us to include more data points in our analyses, and also to avoid problems with using species as a unit for estimating palaeobiodiversity^56^. This is especially pertinent to analyses of the pseudosuchian fossil record, where certain parts of the tree have been the focus of intensive taxonomic work at the species level (for example, Thalattosuchia^57^), whereas other clades are composed primarily of monospecific genera (for example, Notosuchia^26^), and some groups have received relatively little attention (for example, Tomistominae^47^). Despite this issue, fluctuations in the numbers of genera and species are near-identical in both the marine and non-marine realms (Supplementary Fig. 1). The sole exception to this pattern is that there is a continued increase in non-marine species numbers in our most recent time bin (Supplementary Fig. 1), in contrast to the decline that occurs in genera (Fig. 1; Supplementary Fig. 1). We also plotted ratios of non-marine species to genera through time, which shows no significant trend (Supplementary Fig. 2). However, the Neogene differs from earlier periods in pseudosuchian history in having high species to genus ratios (Supplementary Fig. 2). We suggest that these deviations in the Neogene reflect the relative ease of recognizing modern species in the recent fossil record, with most living species belonging to just a small number of genera, and is therefore best considered a ‘Pull of the Recent' type effect.

Extant genera, especially *Alligator* and *Crocodylus*, have been used as ‘wastebasket taxa' for indeterminate or non-referable fossil species. Therefore, we modified occurrences of *Crocodylus* and *Alligator* before analysis, as explained in the Supplementary Methods. We also reviewed Cretaceous thalattosuchian occurrences, to help constrain marine crocodylomorph biodiversity over the J/K boundary (Supplementary Methods).

The resultant pseudosuchian data set was subdivided into non-marine (terrestrial plus freshwater; Supplementary Data 1) and marine taxa (Supplementary Data 2) using an amended version of a list of the names of Mesozoic–Ypresian tetrapod taxa presented in Benson *et al.* (in review). Environmental assignment (marine versus non-marine) for post-Ypresian taxa was based primarily on facies data recorded in the PaleoDB and information presented in refs 16, 28, 46. Vélez-Juarbe *et al.*^46^ demonstrated the marine affinities of several late Oligocene–Neogene gavialoids, including *Aktiogavialis*, *Piscogavialis* and *Siquisiquesuchus*; these were omitted from the marine crocodylomorph data set of Martin *et al.*^16^ without comment, but are incorporated as marine taxa here. Although some stratigraphically older species currently included within *Tomistoma* were probably marine^47^, most occurrences (including the extant species *T*. *schlegelii*) are non-marine, or their environments are unknown; as such, here we treat *Tomistoma* as non-marine.

### Subsampling protocol

Pseudosuchian genera were assigned to approximately equal-length (9 million years) stratigraphic time bins (Supplementary Table 1 and Supplementary Data 3). Although there is variation in time bin duration, there is no significant trend of interval duration through time (Supplementary Fig. 3). We applied equal coverage or ‘shareholder quorum' subsampling (SQS)^58^ to ameliorate the effects of uneven sampling and to reconstruct temporal patterns in past biodiversity. SQS tracks the ‘coverage' of each subsampling pool represented by the taxa that have been drawn^58^. Coverage is the sum of the proportional frequencies of the taxa sampled in each time bin, and coverage of observed data is modified to estimate the coverage of the real taxon distribution for each sample pool. This is achieved by multiplying coverage of the observed data by Good's *u*: the proportion of occurrences representing non-singleton taxa^58^,^59^. Each interval can therefore only be subsampled to a maximum quorum level (i.e. amount of coverage) equal to Good's *u* for that interval, meaning fewer time intervals/geographical regions can be subsampled at higher quorum levels. We used a quorum level of 0.4 for reported analyses, a level which recovers similar patterns to those at higher quorum levels based on marine invertebrate data sets^58^,^59^, and unreported analyses of our pseudosuchian data. The substantial advantage of SQS over other subsampling methods, such as classical rarefaction, is that it is robust to the tendency of those methods to ‘flatten out' biodiversity curves^58^. It has been suggested that SQS can remove genuine biodiversity signals when they are driven by environmental variables that jointly drive geological sample availability^45^. This does not seem to be the case in our data, as the correlations of environmental variables with subsampled biodiversity estimates are stronger than those with face value biodiversity counts (Tables 1 and 2). Furthermore, independent data on pseudosuchian biodiversity in the northern temperate regions, from North America and from Europe, yield highly congruent subsampled biodiversity curves. This is consistent with the effective estimation of a shared underlying biodiversity pattern, although we acknowledge that the adequacy of the SQS method would benefit from more detailed investigation, building on simulation studies^60^.

In determining Good's *u*, singleton taxa were defined based on occurrences within collections^61^, rather than publications^58^,^59^. Entire fossil collections, containing lists of species occurrences, were drawn^62^. Because poorly studied spatiotemporal regions could appear well sampled for stochastic reasons, returning spuriously low subsampled biodiversity estimates, time bins with fewer than six publications were excluded from our analyses. Whenever a collection corresponding to a new publication was drawn, subsequent collections were drawn from that publication only until all or three collections from that publication had been sampled^61^. Results are based on 1,000 subsampling trials. The PERL script used for implementing SQS is provided in Supplementary Data 4, and was written and provided by J. Alroy.

Patterns in non-marine and marine taxa were analysed separately, to avoid problems with sampling from heterogeneous environments^58^,^59^. Marine pseudosuchian genera, which have wide geographic distributions, were analysed as a global data set. However, high levels of endemism are evident in non-marine genera, which were therefore analysed across a set of continental regions (Africa, Asia, Australasia, Europe, North America and South America) representing regional biotas of approximately equal geographic spread. For each continental region, we selected countries that were well-sampled and cohesive through geological time, and therefore valid for deep time analyses. We also attempted to make the geographic spread of data relatively even among continents by excluding far-outlying countries (Supplementary Methods and Supplementary Table 2).

In addition to producing sub-sampled terrestrial and marine biodiversity curves through time, we also analysed the palaeolatitudinal distribution of terrestrial biodiversity. This was implemented through plots of subsampled regional biodiversity against the regional palaeolatitudinal centroid, as well as plots of subsampling curves within time bins (Fig. 3 and Supplementary Fig. 4). Plots of the palaeolatitudinal spread of all pseudosuchian and all tetrapod occurrences through time were also produced for non-marine (Fig. 4a) and marine taxa (Supplementary Fig. 5). Collections in the Paleobiology Database are assigned present-day coordinates and geological ages. These two pieces of information are combined with palaeogeographic rotation models provided by C. Scotese (http://www.scotese.com) to obtain reconstructed palaeogeographic positions for each occurrence.

### Correlation of biodiversity with palaeotemperature and sea level

We compared subsampled genus biodiversities at a quorum of 0.4 to *δ*^18^O palaeotemperature proxies and sequence stratigraphic eustatic sea level estimates in two sets of analyses: (1) terrestrial pseudosuchian biodiversity in North America and Europe were each compared to the Zachos *et al.*^17^ compendium of benthic foraminifera isotopic values (Supplementary Data 5), which spans the latest Maastrichtian to Cenozoic; and (2) global marine pseudosuchian genus biodiversity was compared to the eustatic sea level estimates of Miller *et al.*^18^ (Supplementary Data 6) and *δ*^18^O from the Prokoph *et al.*^40^ compendium (Supplementary Data 7), which includes Jurassic–Recent temperate palaeolatitudinal sea surface isotopic values from a range of marine organisms, adjusted for vital effects.

Although a contentious issue in crocodylomorph phylogeny, we follow the most recent placement of Thalattosuchia as a basal clade outside of Crocodyliformes^63^, rather than within Neosuchia (for example, ref. 26). Consequently, we consider crocodylomorphs to have independently become adapted to marine life in the Jurassic (Thalattosuchia) and Cretaceous (pholidosaurids, dyrosaurids and eusuchians), representing separate temporal and evolutionary replicates that are characterised by distinct groups with possible different biodiversity dynamics. We therefore also analysed relationships between marine biodiversity and climatic variables including a binary variable denoting ‘1' for Jurassic–Hauterivian (mid-Early Cretaceous) intervals and ‘2' for stratigraphically younger intervals. Supplementary Figure 6 shows the palaeotemperature and sea level curves with the weighted means used in our time series regressions. Supplementary Fig. 7 shows plots of subsampled marine biodiversity versus *δ*^18^O and sea level, with and without the application of first differencing.

The similarity of these independent isotopic databases^17^,^40^ for the overlapping portion of geological time suggests that both capture broad patterns of global climate change. Martin *et al.*^16^ compared Jurassic–late Eocene marine crocodylomorph biodiversity with a sea surface temperature (SST) curve established from *δ*^18^O values of fish teeth from the Western Tethys. One potential problem with this method is that the fish teeth are from a variety of different species and genera, with Lécuyer *et al.*^64^ noting that species-specific differences in fractionation of *δ*^18^O can occur. In addition, there might be differences between the isotopic fractionation that occurs between phosphate and water, and that which takes place in the fish teeth^64^. Despite these potential issues, their SST curve broadly follows the *δ*^18^O curves of Prokoph *et al.*^40^ and Zachos *et al.*^17^, suggesting that the overall pattern between them is congruent. However, the benthic *δ*^18^O dataset for deep sea palaeotemperatures of Zachos *et al.*^17^ is much better resolved than that of the SST curve, and the Prokoph *et al.*^40^ data set spans a larger time interval. Consequently, we consider these two datasets^17^,^40^ better suited to testing for a correlation between palaeotemperature and biodiversity than the SST curve^16^,^64^. Time-weighted mean values of each of these two data sets were calculated and used in the regression analyses below.

Statistical comparison was made using time series approaches, specifically generalised least squares (GLS) regression incorporating a first-order autoregressive model (for example, refs 22, 65, 66), and implemented in the R package nlme, using the gls() function^67^. This estimates the strength of serial correlation in the relationship between variables using maximum likelihood during the regression model-fitting process, correcting for the non-independence of adjacent points within a time series. We compared the results to those of ordinary least squares regression using untransformed data, which assumes serial correlation=0. Because intervals lacking marine pseudosuchians, and intervals that did not meet our quorum level due to data deficiency were excluded, our regression analyses ask whether pseudosuchian diversity was correlated to environmental variables when pseudosuchians were present at all.

All analyses were performed in R version 3.0.2 (ref. 68) and using a customized PERL script provided by J. Alroy. Additional information is provided in the Supplementary Methods.

## Additional information

**How to cite this article:** Mannion, P. D. *et al.* Climate constrains the evolutionary history and biodiversity of crocodylians. *Nat. Commun.* 6:8438 doi: 10.1038/ncomms9438 (2015).

## Supplementary Material

Supplementary Information Supplementary Figures 1-7, Supplementary Tables 1-2, Supplementary Methods and Supplementary References

Supplementary Data 1 Non-marine pseudosuchian data

Supplementary Data 2 Marine pseudosuchian data

Supplementary Data 3 Time intervals table

Supplementary Data 4 PERL script for SQS (provided by John Alroy)

Supplementary Data 5 Zachos *et al.* (2008) palaeotemperature proxy isotope data

Supplementary Data 6 Miller *et al.* (2005) sea level data

Supplementary Data 7 Prokoph *et al.* (2008) palaeotemperature proxy isotope data

## Figures and Tables

**Table 1:** Results of generalised least squares regression of log_10_ subsampled non-marine genus biodiversity (quorum=0.4) on the ***δ*** ^18^O palaeotemperature proxy of Zachos *et al.* 17

**Environment**	**Region**	**Palaeotemp.**	**N**	**GLS**			**OLS (untransformed)**			**OLS (fd)**		
				**Phi**	**Interc.**	**Slope**	**Intercept**	**Slope**	**R**^2^	**Interc.**	**Slope**	**R**^2^
Continental	North America	Zachos	9	−0.11	0.42	−0.13** (0.023)	0.41	−0.13** (0.041)	0.39	−0.03	0.03 (0.853)	−0.16
Continental	Europe	Zachos	6	−0.86	1.14	−0.50** (<0.001)	1.09	−0.47** (0.003)	0.89	0.12	−0.83** (0.038)	0.75

‘Palaeotemp.' gives the source of *δ*^18^O data; ‘GLS' denotes generalised least squares regression incorporating a first-order autoregressive covariance model; OLS denotes ordinary least squares regression; ‘fd' indicates that first-differencing was applied to the input data series; phi is the serial correlation coefficient^70^; ‘Intercep.' is the y-intercept; R^2^ is the adjusted R^2^ of ordinary least squares regression, and comparable R^2^ values cannot be computed for generalise least squares. Notes: **significant at alpha=0.05.

**Table 2:** Results of generalised least squares regression of log_10_ subsampled marine genus biodiversity (quorum=0.4) on the ***δ*** ^18^O palaeotemperature proxy of Prokoph *et al.* 40 and estimated sea level^18^.

**Environment**	**Region**	**Independent variable**	**N**	**GLS**			**OLS (untransformed)**		
				**Phi**	**Int.**	**Slope**	**Int.**	**Slope**	**R**^2^
*Subsampled.*									
Marine	Global	Prokoph *δ*^18^O	12	0.68	0.26	−0.05 (0.227)	0.32	−0.03 (0.539)	-0.06
Marine	Global	Prokoph *δ*^18^O	12	0.88	0.25	−0.05 (0.188)	0.31	−0.03 (0.581)	-0.17
		+phase†				−0.09 (0.503)		0.003 (0.972)	
Marine	Global	Sea level	12	0.47	0.32	0.001 (0.159)	0.33	0.002 (0.114)	0.15
Marine	Global	Sea level	12	−0.01	0.46	0.005** (0.002)	0.46	0.005** (0.002)	0.61
		+phase†				−0.25** (0.006)		−0.249** (0.006)	
									
*Counted.*									
Marine	Global	Prokoph *δ*^18^O	18	0.57	0.64	0.047 (0.500)	0.67	0.067 (0.373)	-0.01
Marine	Global	Prokoph *δ*^18^O	18	0.52	0.87	0.066 (0.331)	0.91	0.093 (0.184)	0.19
		+phase†				−0.302 (0.155)		−0.311** (0.042)	
Marine	Global	Sea level	18	0.72	0.60	0.004 (0.172)	0.62	<0.000 (0.978)	-0.06
Marine	Global	Sea level	18	0.54	0.89	0.005* (0.068)	0.96	0.006** (0.047)	0.30
		+phase†				−0.439* (0.050)		−0.545** (0.008)	

‘GLS' denotes generalised least squares regression incorporating a first-order autoregressive covariance model; OLS denotes ordinary least squares regression; ‘fd' indicates that first-differencing was applied to the input data series; phi is the serial correlation coefficient^70^; ‘Int.' is the y-intercept; R^2^ is the adjusted R^2^ of ordinary least squares regression, and comparable R^2^ values cannot be computed for generalised least squares. ; **significant at alpha=0.05 (significance).

^†^including a binary variable indicating the Jurassic–Early Cretaceous marine radiation as “1” and the Late Cretaceous–Cenozoic marine radiation as ‘2'
