# Supplementary material for: Climate constrains the evolutionary history and biodiversity of crocodylians
Source: Nat Commun. 2015 Sep 24;6:8438. doi: 10.1038/ncomms9438 (PMC4598718; doi:10.1038/ncomms9438)
Supplement: Supplementary Information — Supplementary Figures 1-7, Supplementary Tables 1-2, Supplementary Methods and Supplementary References [file ncomms9438-s1.pdf]

Supplementary Figures

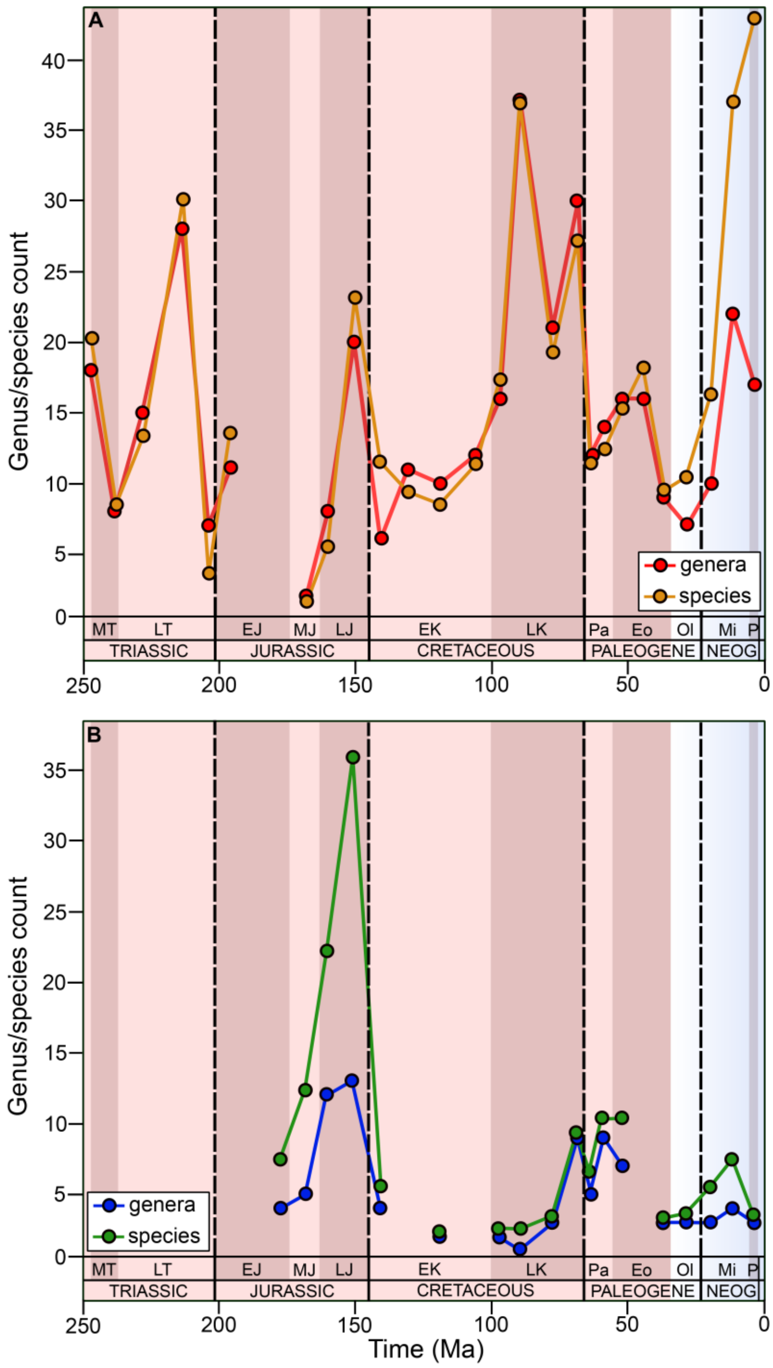

Supplementary Figure 1. Global raw counts of pseudosuchian genera and species through the last 250 million years. (A) Non-marine genera (red line) and species (brown line). (B) Marine genera (blue line) and species (green line). Note that time bins in which numbers of genera exceed numbers of species reflect the presence of specifically-indeterminate occurrences that are diagnostic at the genus level.

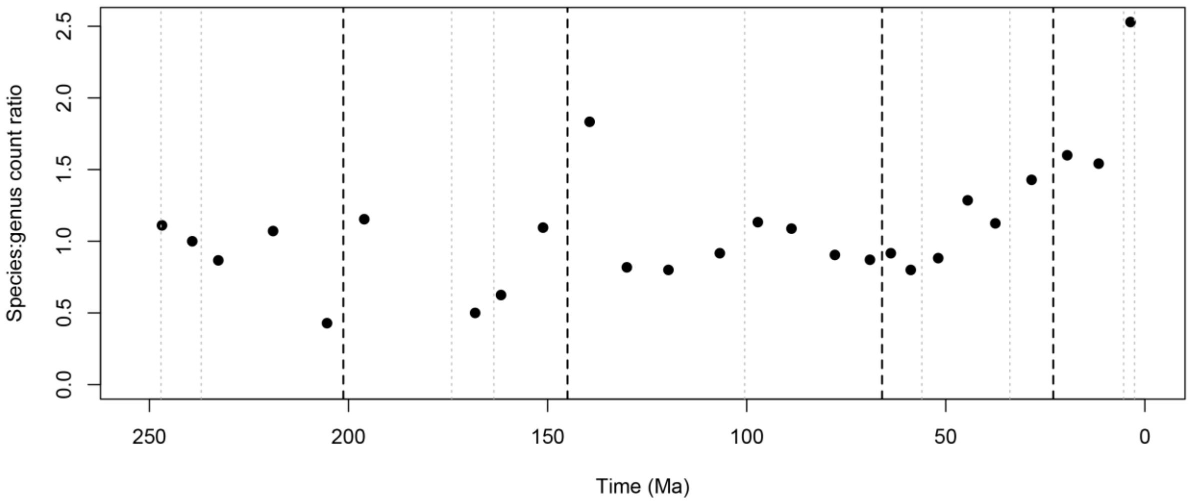

Supplementary Figure 2. Species to genus count ratios through the last 250 million years, showing no significant trend (full time series: p = 0.019; R^2 = 0.1749; time series excluding Neogene: p = 0.366; R^2 = -0.007). R^2 is the adjusted R^2 of ordinary least squares regression.

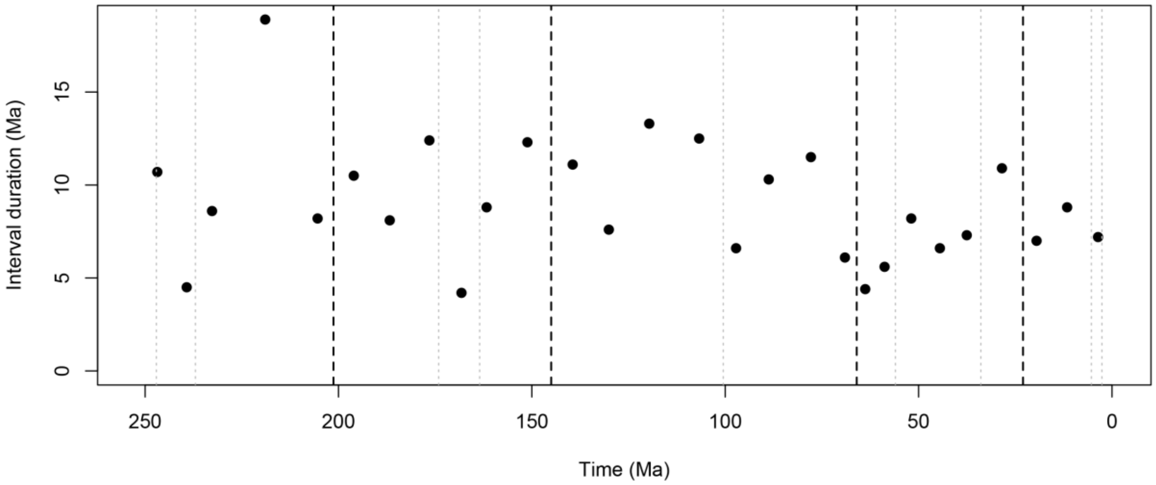

Supplementary Figure 3. Time bin interval durations used in this study, showing no significant trend of interval duration through time (p = 0.161; R^2 = 0.039).

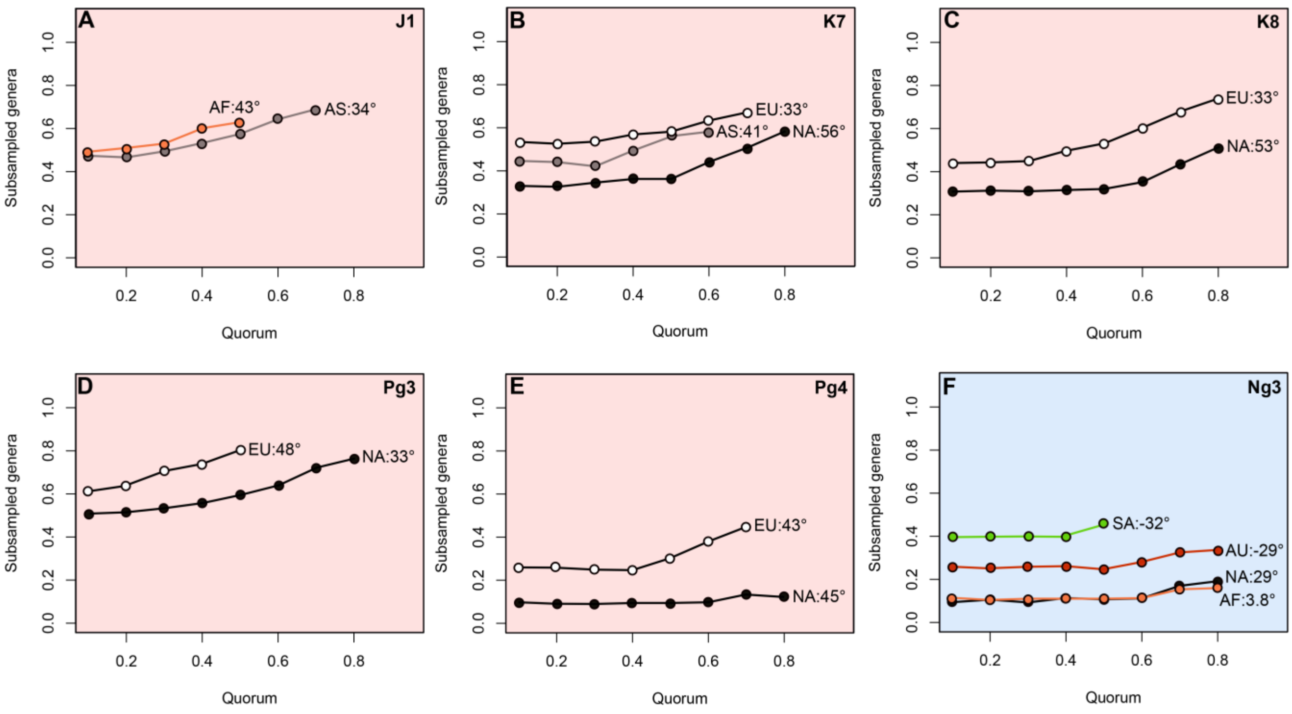

Supplementary Figure 4. Additional $\log_{10}$-transformed regional subsampling curves for non-marine genera. (A) Early Jurassic (J1) subsampling curves. (B) Late Cretaceous (K7) subsampling curves. (C) Late Cretaceous (K8) subsampling curves. (D) Middle Eocene (Pg3) subsampling curves. (E) Late Eocene (Pg4) subsampling curves. (F) Late Neogene (Ng3) subsampling curves.

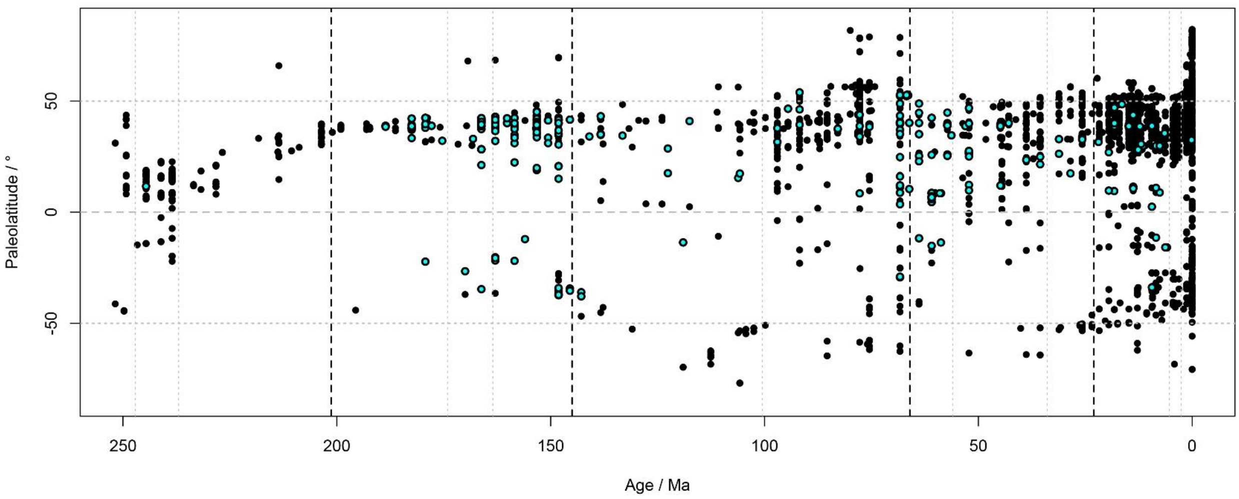

Supplementary Figure 5. Palaeolatitudinal distribution of all marine pseudosuchian (blue circles) and all marine tetrapod (black circles) occurrences through time.

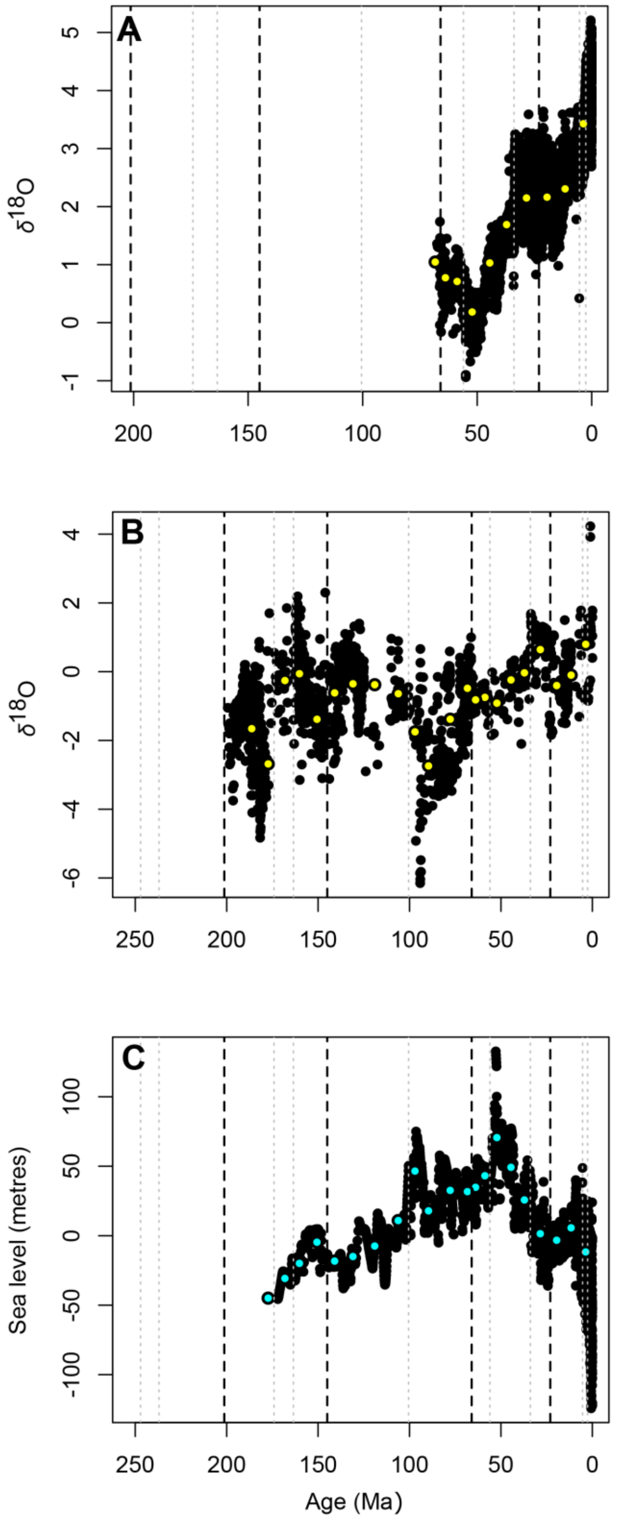

Supplementary Figure 6. Palaeotemperature and sea level through time. (A) Palaeotemperature ($\delta^{18}O$) plot based on Zachos *et al.*[1] with weighted means (yellow circles). (B) Palaeotemperature ($\delta^{18}O$) plot based on Prokoph *et al.*[2] with weighted means (yellow circles). (C) Sea level (metres) plot based on Miller *et al.*[3] with weighted means (blue circles).

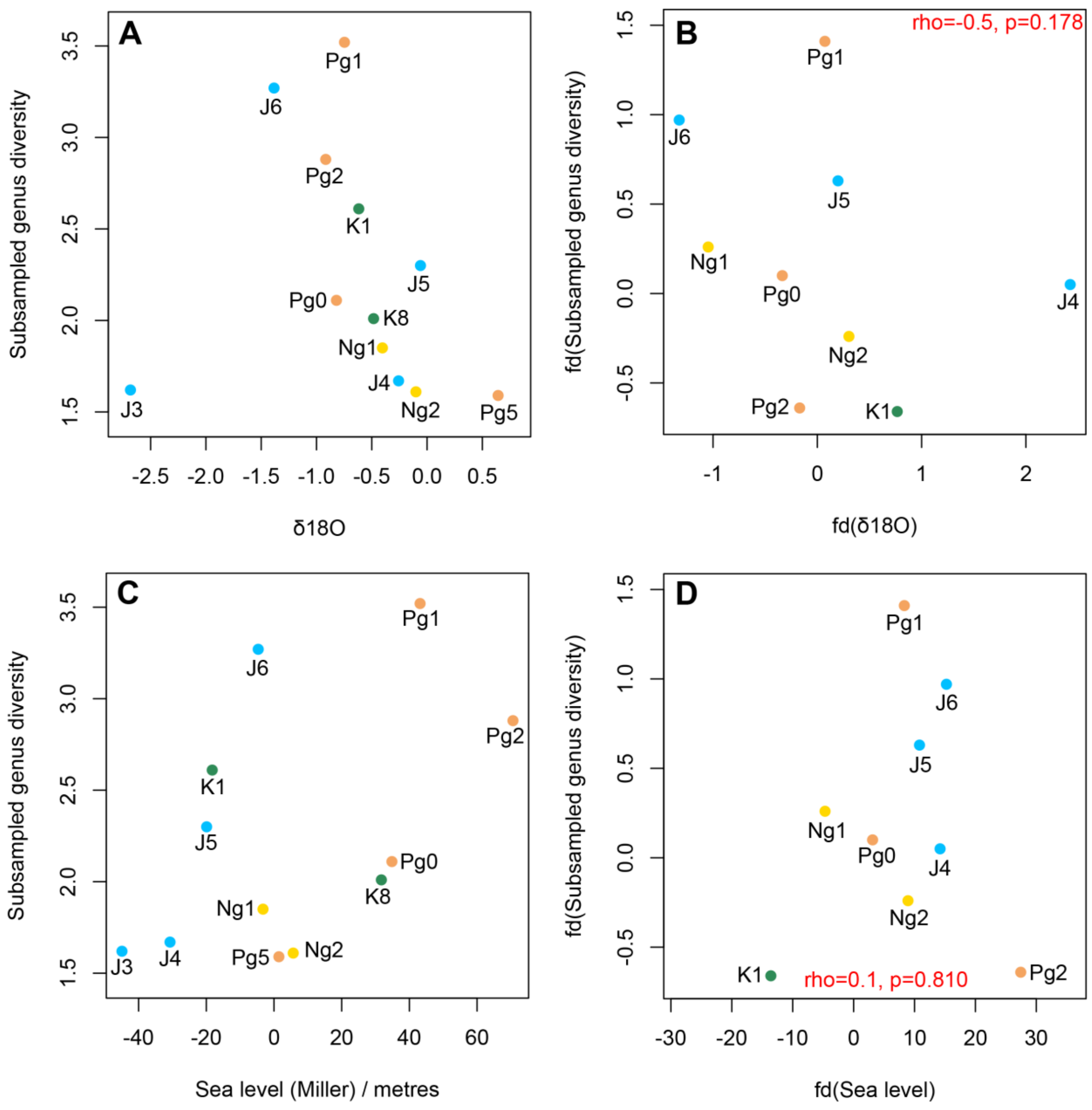

Supplementary Figure 7. Subsampled marine biodiversity versus extrinsic factors. (A) Biodiversity versus δ^{18}O (from Prokoph *et al.*[2]). (B) Biodiversity versus δ^{18}O (from Prokoph *et al.*[2]) using first differences. (C) Biodiversity versus sea level[44]. (D) Biodiversity versus sea level (from Miller *et al.*[3]) using first differences. Correlation statistics are only shown for the transformed (first differenced) data series.

Supplementary Tables

Age of base	Abbreviation	Time interval	Included stages
7.2	Ng3	Late Miocene–Pleistocene	Messinian, Zanclean, Piacenzian, Gelasian
16.0	Ng2	Middle–late Miocene	Langhian, Serravallian, Tortonian
23.0	Ng1	Early Miocene	Aquitanian, Burdigalian
33.9	Pg5	Oligocene	Rupelian, Chattian
41.2	Pg4	Late Eocene	Bartonian, Priabonian
47.8	Pg3	Middle Eocene	Lutetian
56.0	Pg2	Early Eocene	Ypresian
61.6	Pg1	Middle–late Paleocene	Selandian, Thanetian
66.0	Pg0	Early Paleocene	Danian
72.1	K8	Late Cretaceous	Maastrichtian
83.6	K7	Late Cretaceous	Campanian
93.9	K6	Late Cretaceous	Turonian, Coniacian, Santonian
100.5	K5	Late Cretaceous	Cenomanian
113.0	K4	Early Cretaceous	Albian
126.3	K3	Early Cretaceous	Aptian
133.9	K2	Early Cretaceous	Hauterivian, Barremian
145.0	K1	Early Cretaceous	Berriasian, Valanginian
157.3	J6	Late Jurassic	Kimmeridgian, Tithonian
166.1	J5	Middle Jurassic	Callovian, Oxfordian
170.3	J4	Middle Jurassic	Bajocian, Bathonian
182.7	J3	Early–Middle Jurassic	Toarcian, Aalenian
190.8	J2	Early Jurassic	Pliensbachian
201.3	J1	Early Jurassic	Hettangian, Sinemurian
209.5	Tr5	Late Triassic	Rhaetian
228.4	Tr4	Late Triassic	Norian
237.0	Tr3	Late Triassic	Carnian
241.5	Tr2	Middle Triassic	Ladinian
252.2	Tr1	Early–Middle Triassic	Induan, Olenekian, Anisian

Supplementary Table 1. Composite, approximately 9 million year time bins used in the present study. Absolute values are given to one decimal place. Abbreviations: Tr=Triassic; J=Jurassic; K=Cretaceous; Pg=Paleogene; Ng=Neogene.

**Region**	**Included countries**
North America	United States, Canada, Mexico
Europe	United Kingdom, France, Germany, Italy, Switzerland, Spain, Belgium, Germany, Romania, Sweden, Czech Republic, Denmark, Slovenia, Norway, Luxembourg, Netherlands, Ukraine, Hungary, Austria, Poland, Croatia, Portugal
Asia	China, Mongolia, South Korea, Russian Federation, North Korea
South America	Argentina, Chile, Brazil, Bolivia, Colombia, Uruguay, Peru, Venezuela
Africa	Zambia, Namibia, Zimbabwe, Mali, Angola, Ethiopia, Cameroon, Malawi, Senegal, Tanzania, Eritrea, Sudan, Kenya, Libya, Niger, Tunisia, Algeria, Lesotho, Morocco, South Africa
Australasia	Australia

Supplementary Table 2. Countries included in our contiguous continental regions.

Supplementary Methods

Modifications to fossil occurrences of extant genera. The database contains numerous fossil occurrences and species referred to extant pseudosuchian genera. Whereas many of these are considered genuine early occurrences of living taxa, others are not. *Crocodylus* has been

hypothesized to have evolved in the late Miocene based on both molecular[4–6] and morphological work, including the oldest known fossil occurrences that can be referred to the modern genus[7–10]. Dozens of fossil species have been referred to *Crocodylus*, but most of these have since been allocated to other genera or are too fragmentary to recognize as valid taxa[e.g.7,10–13]. Five extinct species with fossil records are considered valid and likely to belong to *Crocodylus*, or are at least close to this radiation: *C. anthropophagus, C. checchiai, C. falconensis, C. palaeindicus,* and *C. thorbjarnarsoni*[14–16].

Six taxa (*C. acer, C. affinis*, *C. clavirostris*, *C. depressifrons*, *C. gariepensis*, and *C. megarhinus*) originally described as species of *Crocodylus*, but no longer considered referable to the genus, are widely regarded as taxonomically distinct[7,11,13,14,16,17] but have yet to receive a new name. However, some of these might represent species of existing genera, rather than distinct genera. The North American taxon *C. acer* has been recovered as the sister taxon to *Brachychampsa*[e.g.16] and thus could potentially be included within that genus. However, whereas *Brachychampsa* is restricted to the Late Cretaceous–early Paleocene of North America, *C. acer* is known from the early Eocene. As such, there is no temporal overlap between the two, and thus we consider *C. acer* a distinct genus for the purposes of our analyses, without risk of within-time-bin biodiversity inflation. *C. affinis* and *C. depressifrons* are known from the Eocene of North America and Europe respectively, and both have been regarded as closely related to, or even referable to, the basal crocodyloid *Asiatosuchus*[e.g.17]. However, whereas *C. depressifrons* is found in deposits that are spatiotemporally contemporaneous with *Asiatosuchus*, and thus its inclusion might artefactually inflate European diversity, no North American material has been referred to *Asiatosuchus*. As such, although *C. affinis* could potentially belong to *Asiatosuchus*, consideration of it as a distinct genus will not overinflate our reconstruction of North American biodiversity. *C. clavirostris* was found in deposits that are approximately contemporaneous with those of *Eosuchus minor*, and the two might be closely related[18], as such, we do not regard *C. clavirostris* as a distinct genus. *C. gariepensis* has been recovered in a clade with *Mecistops* and *Euthecodon*[13], and overlaps geographically and

temporally with the latter. Consequently, it could be a species of *Euthecodon* and accordingly we do not include it as a distinct genus. Lastly, *C. megarhinus* is recovered near the base of the clade that includes extant Crocodylinae (*Crocodylus*, *Mecistops* and *Osteolaemus*), and does not appear referable to any existing genus[e.g.16]. Therefore, here we regard *C. megarhinus* as a distinct genus. In summary, we recognize *C. acer*, *C. affinis* and *C. megarhinus* as distinct genera for the purposes of our analyses, and exclude *C. clavirostris*, *C. depressifrons* and *C. gariepensis*.

Several putative species of *Crocodylus* based on fragmentary material have occurrences in the PaleoDB, but lack recorded taxonomic opinion data: we exclude *C. blavieri*, *C. humilis*, *C. kutchensis*, *C. proavus*, *C. selaslophensis* and *C. vetustus* as *nomina dubia* that do not represent distinct taxa. Based on the proposed timing of the evolution of *Crocodylus* (see above), we exclude specifically indeterminate pre-late Miocene occurrences referred to this genus (i.e. *Crocodylus* sp.). A small number of fragmentary occurrences from the Neogene of Africa have been tentatively referred to the extant genera *Mecistops* and *Osteolaemus*[19,20]. Although these assignments might ultimately prove incorrect, they are in keeping with the expected timing of the evolution of these two taxa[e.g.4], and as such are retained herein.

All *Alligator* species with occurrences in the PaleoDB (the extinct taxa *A. luicus*, *A. mefferdi*, *A. olseni*, *A. prenasalis*, *A. thomsoni*, as well as the extant species *A. mississippiensis* and *A. sinensis*) are considered valid species of this genus[21,22]. Paleocene occurrences referred to *Alligator*[23] are excluded because of their fragmentary and non-diagnostic nature. All occurrences of *Caiman*, *Melanosuchus* and *Paleosuchus* in the PaleoDB are restricted to the Neogene and Quaternary[e.g.15,21,24–28], and are therefore accepted as valid.

Several extinct species of *Gavialis*, along with specifically indeterminate remains, are known from the Neogene of the Indian subcontinent and southeast Asia[29–31]. The oldest known occurrence of *Gavialis* is from the early Miocene of Pakistan[32], which is stratigraphically congruent with the ages of closely related taxa[e.g.33], and with the approximate timing of a proposed split from

Tomistoma, according to molecular studies[e.g.34,35]. As such, we make no changes to *Gavialis* in our dataset.

As with other extant crocodylian taxa, numerous fossil species have been assigned to *Tomistoma*, although several have been allocated to new genera or regarded as non-diagnostic (see Piras *et al.*[36] for a review). Currently, relationships within Tomistominae are poorly resolved, especially regarding whether any fossil species can be included in *Tomistoma*[36–38]. Whereas most of these putative *Tomistoma* occurrences are Neogene, and thus in keeping with the proposed timing of the evolution of the genus[e.g.35], two species (*T. petrolica* and *T. tandoni*) have been described from Eocene deposits[36]. As some of these species are likely distinctive taxa (e.g. *T. cairense*, *T. lusitanicum*, *T. petrolica*), even if not referable to *Tomistoma*[36,38], and the group is in need of detailed taxonomic revision, we do not exclude any occurrences currently referred to *Tomistoma* from our analyses.

Cretaceous thalattosuchians. Thalattosuchia has been the subject of numerous recent taxonomic revisions[e.g.39–43], resulting in the recognition of only five genera unambiguously present in the Cretaceous. *Cricosaurus* is known from remains from the Berriasian of Argentina and the Valanginian of western Europe[40], including material originally referred to '*Enaliosuchus*' and *Geosaurus*[43]. Two Berriasian Argentinean localities have yielded remains of *Dakosaurus*[44]. *Geosaurus* and *Neustosaurus* are present in the Valanginian–Hauterivian of France[43]. *Peipehsuchus* was identified as a thalattosuchian[42] and is known from the Tzeliuching Formation of China, but the age of this stratigraphic unit cannot currently be constrained more accurately than Early Cretaceous.

Numerous Early Cretaceous remains have been referred to *Machimosaurus*, but all of these have either been refuted, or their affinities remain uncertain[41]. Late Cretaceous referrals from Sudan[45] and Brazil[46] are fragmentary and their affinities remain unsubstantiated. An occurrence of the otherwise Jurassic taxon *Teleosaurus* is apparently present in the Valanginian of the UK[47], but this specimen does not seem to have been mentioned in the recent literature, including recent reviews of

the UK fossil record[e.g.48,49], and its affinities remain unclear. Consequently, we restrict *Machimosaurus* and *Teleosaurus* to the Jurassic, in keeping with the view that teleosauroids went extinct at the Jurassic/Cretaceous boundary[40,50].

Time-binning scheme. Our scheme of time bins comprises approximate 9 myr bins following the Standard European stages and absolute dates provided by Gradstein *et al.*[51], and is shown in Supplementary Table 1. Occurrences were assigned to a time bin only if their stratigraphic age uncertainty was entirely contained within that bin. The exception to this is pseudosuchian occurrences from the Brazilian Late Cretaceous Adamantina Formation. Currently, fifteen pseudosuchian genera (all belonging to Notosuchia, with several taxa represented by multiple occurrences) are known from this stratigraphic unit, which was deposited at subtropical palaeolatitudes ranging from 24–28°S[52,53]. However, there is little consensus on the age of the formation[53], with some authors proposing a Turonian–Santonian age, based on ostracods[54], whereas other have argued for a Campanian–Maastrichtian age, based on vertebrate fossils[e.g.55,56]. As a result of the high biodiversity of this formation, coupled with its geographical position, we incorporate the Adamantina Formation and its constituent pseudosuchian taxa into our analyses by arbitrarily assigning it to our K6 (Turonian, Coniacian, Santonian) time bin (see Supplementary Table 1), on the basis that the ostracod data[54] is more likely to be a reliable indicator of its age.

Spatial-binning scheme. Continental regions were selected by targeting contiguous, well-sampled areas of approximately comparable geographic spread (Supplementary Table 2). For example, although peripheral sub-regions such as Cuba and Greenland are technically parts of North America by cartographical conventions, they are geographically distant to, and poorly sampled compared to, the core regions of North America (United States, Canada, Mexico). Including these peripheral regions has the effect of adding a small number of occurrences of primarily singleton taxa to the sampling pool of North America. The addition of singletons causes the core region to appear less

well sampled than in fact it is, and thereby inflates subsampled diversity estimates. A similar protocol was used for other regions, e.g. Japan and southern Asian countries were excluded from our Asian region.

Supplementary References

1. Zachos, J. C., Dickens, G. R. & Zeebe, R. E. An early Cenozoic perspective on greenhouse warming and carbon-cycle dynamics. *Nature* **451,** 279–283 (2008).
2. Prokoph, A., Shields, GA & Veizer, J. Compilation and time-series analysis of a marine carbonate $\delta^{18}O$, $\delta^{13}C$, $^{87}Sr/^{86}Sr$ and $\delta^{34}S$ database through Earth history. *Earth-Sci. Rev.* **87,** 113–133 (2008).
3. Miller, K. G. *et al.* The Phanerozoic record of global sea level change. *Science* **310,** 1293–1297 (2005).
4. Oaks, J. R. A time-calibrated species tree of Crocodylia reveals a recent radiation of true crocodiles. *Evolution* **65,** 3285–3297 (2011).
5. Meganathan, P. R., Dubey, B., Batzer, M. A., Ray, D. A. & Haque, I. Molecular phylogenetic analyses of genus *Crocodylus* (Eusuchia, Crocodylia, Crocodylidae) and the taxonomic position of *Crocodylus porosus*. *Mol. Phylogenet. Evol.* **57,** 393–402 (2010).
6. Meredith, R. W., Hekkala, E. R., Amato, G. & Gatesy, J. A phylogenetic hypothesis for *Crocodylus* (Crocodylia) based on mitochondrial DNA: evidence for a trans-Atlantic voyage from Africa to the New World. *Mol. Phylogenet. Evol.* **60,** 183–191 (2011).
7. Brochu, C. A. Phylogenetic relationships and divergence timing of *Crocodylus* based on morphology and the fossil record. *Copeia* **2000,** 657–673 (2000).
8. Storrs, G. W. in *Lothagam: The Dawn of Humanity in Eastern Africa* (eds Leakey, M. G. & Harris, J. M. (eds.) 137–159 (Columbia Univ. Press, 2003).

9. Delfino, M., Böhme, M. & Rook, L. First European evidence for transcontinental dispersal of *Crocodylus* (late Neogene of southern Italy). *Zool. J. Linn. Soc.* **149,** 293–307 (2007).

10. Delfino, M. & Rook, L. African crocodylians in the Late Neogene of Europe: a revision of *Crocodylus bambolii* Ristori, 1890. *J. Paleontol.* **82,** 336–343 (2008).

11. Norell, M. A. & Storrs, G. W. Catalogue and review of the type fossil crocodilians in the Yale Peabody Museum. *Postilla* **203,** 1–28 (1989).

12. Brochu, C. A. Morphology, relationships, and biogeographical significance of an extinct horned crocodile (Crocodylia, Crocodylidae) from the Quaternary of Madagascar. *Zool. J. Linn. Soc.* **150,** 835–863 (2007).

13. Conrad, J. L. *et al.* New specimens of '*Crocodylus*' *pigotti* (Crocodylidae) from Rusinga Island, Kenya, and generic reallocation of the species. *J. Vertebr. Paleontol.* **33,** 629–646 (2013).

14. Brochu, C. A., Njau, J., Blumenschine, R. J. & Densmore, L. D. A new horned crocodile from the Plio-Pleistocene hominid sites at Olduvai Gorge, Tanzania. *PLoS ONE* **5,** e9333 (2010).

15. Scheyer, T. M. *et al.* Crocodylian diversity peak and extinction in the late Cenozoic of the northern Neotropics. *Nat. Commun.* **4,** 1907 (2013).

16. Brochu, C. A. & Storrs, G. W. A giant crocodile from the Plio-Pleistocene of Kenya, the phylogenetic relationships of Neogene African crocodylines, and the antiquity of *Crocodylus* in Africa. *J. Vertebr. Paleontol.* **32,** 587–602 (2012).

17. Delfino, M. & Smith, T. A reassessment of the morphology and taxonomic status of '*Crocodylus*' *depressifrons* Blainville, 1855 (Crocodylia, Crocodyloidea) based on the Early Eocene remains from Belgium. *Zool. J. Linn. Soc.* **156,** 140–167 (2009).

18. Brochu, C. A. Osteology and phylogenetic significance of *Eosuchus minor* (Marsh, 1870) new combination, a longirostrine crocodylian from the late Paleocene of North America. *J. Paleontol.* **80,** 162–186 (2006).

19. Aoki, R. Fossil crocodilians from the late Tertiary strata in the Sinda Basin, eastern Zaire. *African Study Monographs* **17,** 67–85 (1992).

20. Pickford, M. in *Geology and Palaeobiology of the Albertine Rift Valley, Uganda−Zaire, Vol. II, Palaeobiology* (eds Senut, B. & Pickford, M.) 137–155 (Centre International pour la formation et les échanges Géologiques (CIFEG), Orleans, Occasional Publications, 1994).

21. Brochu, C. A. Phylogenetics, taxonomy, and historical biogeography of Alligatoroidea. *J. Vertebr. Paleontol.* **19,** 9–100 (1999).

22. Snyder, D. Morphology and systematics of two Miocene alligators from Florida, with a discussion of alligator biogeography. *J. Paleontol.* **81,** 917–928 (2007).

23. Erickson, B. R. Crocodilians of the Black Mingo Group (Paleocene) of the South Carolina Coastal Plain. *T. Am. Philol. Assoc.* **88,** 196–214 (1998).

24. Salas-Gismondi R. *et al.* A Miocene hyperdiverse crocodylian community reveals peculiar trophic dynamics in proto-Amazonian mega-wetlands. *Proc. R. Soc. Lond. Ser. B* **282,** 20142490 (2015).

25. Medina, C. J. Crocodilians from the Late Tertiary of northwestern Venezuela: *Melanosuchus fisheri* sp. nov. *Breviora* **438,** 1–14 (1976).

26. Salas-Gismondi, R. *et al.* Middle Miocene crocodiles from the Fitzcarrald Arch, Amazonian Peru. *Cuadernos del Museo Geominero* **8,** 355–360 (2007).

27. Bona, P. & Carabajal, A. P. *Caiman gasparinae* sp. nov., a huge alligatorid (Caimaninae) from the late Miocene of Paraná, Argentina. *Alcheringa* **37,** 462–473 (2013).

28. Fortier, D. C. & Rincón, A. D. Pleistocene crocodylians from Venezuela, and the description of a new species of *Caiman*. *Quatern. Int.* **305,** 141–148 (2013).

29. Lull, R. S. Fossil gavials from North India. *Am. J. Sci.* **242,** 417–430 (1944).

30. Delfino, M. & de Vos, J. A revision of the Dubois crocodylians, *Gavialis bengawanicus* and *Crocodylus ossifragus,* from the Pleistocene *Homo erectus* beds of Java. *J. Vertebr. Paleontol.* **30,** 427–441 (2010).

31. Martin, J. E., Buffetaut, E., Naksri, W., Lauprasert, K. & Claude, J. *Gavialis* from the Pleistocene of Thailand and its relevance for drainage connections from India to Java. *PLoS ONE* **7,** e44541 (2012).

32. Piras, P. & Kotsakis, T. A new gavialid from the Early Miocene of south-eastern Pakistan (Preliminary Report). *Rendiconti Societa Paleontologica Italiana* **2,** 201–207 (2005).

33. Vélez-Juarbe, J., Brochu, C. A. & Santos, H. A gharial from the Oligocene of Puerto Rico: transoceanic dispersal in the history of a non-marine reptile. *Proc. R. Soc. Lond. Ser. B* **274,** 1245–1254 (2007).

34. Janke, A., Gullberg, A., Hughes, S., Aggarwal, R. K. & Arnason, U. Mitogenomic analyses place the gharial (*Gavialis gangeticus*) on the crocodile tree and provide pre-K/T divergence times for most crocodilians. *J. Mol. Evol.* **61,** 620–626 (2005).

35. Willis, R. E., McAliley, L. R., Neeley, E. D. & Densmore III, L. D. Evidence for placing the false gharial (*Tomistoma schlegelii*) into the family Gavialidae: Inferences from nuclear gene sequences. *Mol. Phylogenet. Evol.* **43,** 787–794 (2007).

36. Piras, P., Delfino, M., Favero, L. & Kotsakis, T. Phylogenetic position of the crocodylian *Megadontosuchus arduini* and tomistomine palaeobiogeography. *Acta Palaeontol. Pol.* **52,** 315–328 (2007).

37. Kobayashi, Y., Tomida, Y., Kamei, T. & Eguchi, T. Anatomy of a Japanese tomistomine crocodylian, *Toyotamaphimeia machikanensis* (Kamei et Matsumoto, 1965) from the Middle Pleistocene of Osaka prefecture: the reassessment of its phylogenetic status within Crocodylia. *National Science Museum Monograph* **35,** 1–121 (2006).

38. Brochu, C. A. Systematics and taxonomy of Eocene tomistomine crocodylians from Britain and northern Europe. *Palaeontology* **50,** 917–928 (2007).

39. Pierce, S. E., Angielczyk, K. D. & Rayfield, E. J. Morphospace occupation in thalattosuchian crocodylomorphs: skull shape variation, species delineation and temporal patterns. *Palaeontology* **52,** 1057–1097 (2009).

40. Young, M. T., Andrade, M. B., Cornée, J.-J., Steel, L. & Foffa, D. Re-description of a putative Early Cretaceous "teleosaurid" from France, with implications for the survival of metriorhynchids and teleosaurids across the Jurassic-Cretaceous Boundary. *Annales de Paléontologie* **100,** 165–174 (2014).

41. Young, M. T. *et al.* Revision of the Late Jurassic teleosaurid genus *Machimosaurus* (Crocodylomorpha, Thalattosuchia). *R. Soc. Open Sci.* **1,** 140222 (2014).

42. Jouve, S. The skull of *Teleosaurus cadomensis* (Crocodylomorpha; Thalattosuchia), and phylogenetic analysis of Thalattosuchia. *J. Vertebr. Paleontol.* **29,** 88–102 (2009).

43. Young, M. T. & Andrade, M. B. What is *Geosaurus*? Redescription of *Geosaurus giganteus* (Thalattosuchia: Metriorhynchidae) from the Upper Jurassic of Bayern, Germany. *Zool. J. Linn. Soc.* **157,** 551–585 (2009).

44. Gasparini, Z., Pol, D. & Spalletti, L. A. An unusual marine crocodyliform from the Jurassic-Cretaceous boundary of Patagonia. *Science* **311,** 70–73 (2006).

45. Werner, C. Die kontinentale Wirbeltierfauna aus der unteren Oberkreide des Sudan (Wadi Milk Formation). *Berliner Geowissenschaften Abhandlungen, Reihe E* **13,** 221–249 (1994).

46. Huene, F, von. Verschiedene mesozoische Wirbeltierreste aus Südamerika. *Neues Jahrbuch für Mineralogie, Geologie und Paläontologie, Abteilung A* **66,** 181–198 (1931).

47. Etheridge, R. *Manual of Geology: Theoretical and Practical. Part II. Stratigraphical Geology and Palaeontology* (Charles Griffin & Co., 1885).

48. Benton, M. J. & Spencer, P. S. *Fossil Reptiles of Great Britain* (Chapman & Hall, 1995).

49. Salisbury, S. W. & Naish, D. in *English Wealden Fossils* (ed Batten D. J.) 305–369 (Palaeontological Association, London, Field Guide to Fossils, 2011).

50. Martin, J. E., Amiot, R., Lécuyer, C. & Benton, M. J. Sea surface temperature contributes to marine crocodylomorph evolution. *Nat. Commun.* **5,** 4658 (2014).

51. Gradstein, F. M *et al. The Geologic Time Scale 2012* (Elsevier, 2012).

52. Carvalho, I. S., Gasparini, Z. B., Salgado, L., de Vasconcellos, F. M. & Marinho, T. S. Climate's role in the distribution of the Cretaceous terrestrial Crocodyliformes throughout Gondwana. *Palaeogeogr. Palaeoclimatol. Palaeoecol.* **297,** 252–262 (2010).

53. Pol, D. *et al.* A new notosuchian from the Late Cretaceous of Brazil and the phylogeny of advanced notosuchians. *PLoS ONE* **9,** e93105 (2014).

54. Dias-Brito, D. *et al.* Grupo Bauru: uma unidade continental do Cretáceo no Brasil—concepções baseadas em dados micropaleontológicos, isotópicos e estratigráficos. *Revue de Paleobiologie* **20,** 245–304 (2001).

55. Gobbo-Rodrigues, S. R., Petri, S. & Bertini, R.J. Ocorrências de ostrácodes na Formação Adamantina do Grupo Bauru, Cretáceo Superior da Bacia do Paraná e possibilidades de correlação com depósitos isócronos Argentinos. Parte I—Família Ilyocyprididae. *Acta Geológica Leopoldensia* **23,** 3–13 (1999).

56. Fernandes, L. A. & Coimbra, A. M. Revisão estratigráfica da parte oriental da Bacia Bauru (Neocretáceo). *Revista Brasileira de Geociências* **30,** 717–728 (2000).
